# Restless Genital Syndrome: Case Report of a Rare Disorder from Pakistan

**DOI:** 10.7759/cureus.2619

**Published:** 2018-05-13

**Authors:** Imran Ahmad, Sarah Rashid, Farooq A Rathore

**Affiliations:** 1 Department of Neurology, Bahria University Medical and Dental College, Bahria University, Karachi, PAK; 2 Department of Rehabilitation Medicine, PNS Shifa Hospital, DHA II, Karachi 75500, Pakistan

**Keywords:** pain, genital pain, dopamine agonist (da), dopamine, pakistan, delayed presentation, sleep quality, rare cases, case report, restless leg syndrome

## Abstract

Restless genital syndrome (RGS) is a newly recognized syndrome characterized by difficult to describe genital sensations, including itching, tingling, contractions, and even pain. It can be a source of distress for the patient and may lead to social withdrawal and delayed diagnosis. Many pharmacologic and non-pharmacologic treatment options have been documented in the literature. Dopamine agonists have been shown to be the most effective in symptomatic relief. We present a case of an Asian female with symptoms suggestive of RGS for 11 years before she was diagnosed who responded well to ropinirole. We discuss the pathophysiology and reasons for the delayed diagnosis.

## Introduction

Restless genital syndrome (RGS) is a rare disorder, and only few case reports have been documented in the English biomedical literature [1-8]. The intrusive genital symptoms may be described as irritation, tingling, discomfort, itching, congestion, and pain [1]. The disease can be an immense source of distress and disability for the patients and may lead to social embarrassment and isolation.

In 2001, a syndrome of persistent sexual arousal syndrome (PSAS) was described for the first time in patients without Parkinson’s disease [5]. The clinical criteria for PSAS were defined as an involuntary genital arousal that persists for an extended period (hours to months) and does not go away with one or more orgasms, is unrelated to feelings of sexual desire, is intrusive and unwanted, and is associated with significant distress [3]. The pathogenesis proposed include pelvic vasocongestion, pelvic varices, and neuropathy of the pudendal nerves [6-7]. Hyperesthesia, neuropathy, and allodynia have also been suggested as possible mechanisms for this disorder [7]. In 2009, preexisting restless leg syndrome (RLS) was found in 12 of 18 patients with similar symptoms, and the term restless genital syndrome (RGS) was introduced [5].

Restless genital syndrome can also affect males [2]. There are many similarities between RGS, tardive genital pain, and genital pain in Parkinson’s disease, suggesting a possible dopaminergic mechanism [5]. Currently, RGS is a disorder of somatosensory function rather than a sexual dysfunction, and hence, the need to redefine the clinical criteria [6].

A detailed clinical history is essential for the diagnosis of this disorder, and treatment with dopamine agonists can be beneficial [6]. However, the knowledge about the pathophysiology and treatment for RGS is still evolving, and many non-neurologist physicians might not know of this disorder. This may lead to unnecessary investigations and a delay in the diagnosis.

We describe the case report of a married female with RGS from Pakistan, a low middle-income country (LMIC), which remained undiagnosed for 11 years before a neurology consultation established the correct diagnosis.

## Case presentation

A 44-year-old previously healthy Asian female reported to the neurology clinic with complaints of episodic, persistent, uncomfortable needle-like sensations in her genitalia extending to the anal area and the tip of the coccyx. She had been suffering from these symptoms for approximately 11 years. She had difficulty describing the actual nature of this phenomenon, which, according to her, was more of an irritation and discomfort than actual pain. The discomfort was aggravated during rest and periods of inactivity, particularly at night. It responded briefly to mefenamic acid tablets for four to five hours. She also reported the pain was worse before her monthly menstrual cycle but that sexual activity did not affect her discomfort. Sometimes, the discomfort was intense and would wake her from sleep causing severe discomfort and resulting in difficulty sitting; walking would relieve her symptoms partially. She was better in the morning, but the symptoms appeared again at end of the day. She had multiple gynecology and dermatology consults with no relief or clear diagnosis. She was prescribed antifungal creams, including fluconazole and ketoconazole, topical steroids (betamethasone and hydrocortisone), and mefenamic acid for pain relief. All failed to bring relief. She also consulted a homeopathy practitioner, who labeled her as suffering from a chronic skin disease and prescribed oral and topical medication. She took it for a few months but stopped it due to lack of relief. Ultimately, due to the distress and anguish associated with the condition, lack of response to various treatments, and social embarrassment, she stopped visiting doctors for some time. However, during a recent visit, the consulting gynecologist suspected it was not a dermatological or gynecological problem and referred her for a neurology consult.

**Figure 1:**
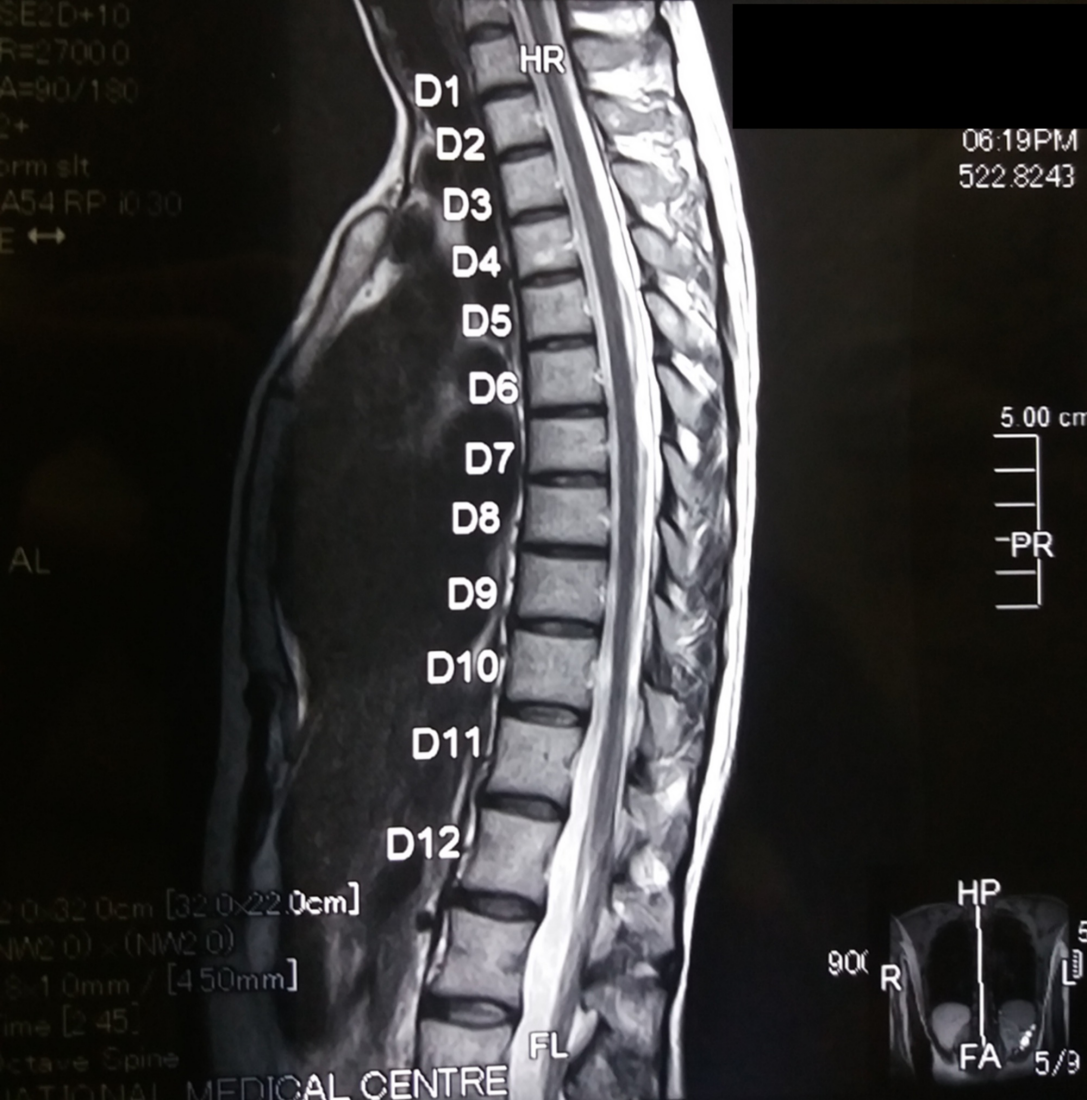
Plain MRI of the thoracolumbar spine did not reveal any pathology MRI: magnetic resonance imaging

There was no history of back pain, sensory symptoms in the legs, urinary incontinence, or neurological disorder, such as Parkinsonism or restless leg disorder. She had a stable and happy marital life of 22 years and had three children. She has used no substance of abuse or recreational drugs. Her neurological and gynecological examinations, as well as magnetic resonance imaging (MRI) of the spine, were normal (Figure 1 ). Similarly, her biochemical profile (including complete blood count, urine analysis, serum urea, and creatinine) and an ultrasound examination of abdomen and pelvis were normal.

Considering her history of circadian rhythm variations in symptoms and aggravation on rest, RGS was considered the most likely cause of her symptoms. After a detailed discussion with the patient, she was prescribed 0.5 mg of ropinirole once at night. It was increased to twice daily after seven days, to which she responded well and reported a dramatic relief of symptoms after 14 days. The dose was increased to 1 mg in the morning and 0.5 mg at night, which led to further improvement. On her third follow-up after two months, she reported taking her medication regularly, which led to almost no episodes of discomfort, with exception of one or two times. The dose of ropinirole was maintained for one month. At the three-month follow-up, she reported only one episode of pain and itching, when she missed one dose of her medicine for a few days; otherwise, she had no complaints and reported no side effects of the medication.

## Discussion

RGS is characterized by potentially exhausting and painful genital sensations that occur spontaneously and might be associated with restless leg syndrome and/or overactive bladder [6]. The pathophysiology of this disorder is not clearly understood. However, researchers have proposed various causes, including neurologic, psychologic, and vascular disorders [7-8]. Our patient had been experiencing persistent painful genital sensations for 11 years, which had disturbed her night sleep. Although her clinical features followed RGS, the diagnosis was delayed, likely due to the lack of an early neurological consult. Her description of worsening of symptoms before her monthly menstrual cycle has also been reported in most female patients with RGS [7]. In this case, she was clinically diagnosed based on her symptoms and negative findings on MRI and hence can be considered as a diagnosis of exclusion.

To date, there have been multiple reports of restlessness in different parts of the body, such as the abdomen, bladder, and genital region, either in isolation or associated with RLS [3]. Around 67% of cases of RGS coexist with RLS [3]. Dopaminergic agonists are used as the first line therapy for RLS; however, they have not been readily used in the treatment of RGS, owing to a poor recognition of the association between the two [3].

The patient was prescribed ropinirole (0.5 mg to 1 mg), which proved to be effective, suggesting its pathophysiology may involve the dopaminergic mechanism. Her diagnosis was made based on described symptoms of RGS in previous research, and the dramatic response to ropinirole gave evidentiary support in confirming our suspicions.

Other treatment options for RGS reported in the literature include electroconvulsive therapy (especially if it is associated with mood symptoms), transcutaneous electrical nerve stimulation, and anti-anxiety medicines, like diazepam, which may provide symptomatic relief [9]. Many cases also have been reported to be treated with dopamine agonists (e.g., pramipexole, ropinirole, rotigotine) that proved beneficial in the treatment of RGS [10].

The patient suffered because of her condition for a long time before she was finally diagnosed as RGS and responded well to ropinirole. Mostly, negative emotions and distress are associated with this condition. During her illness, she had consulted many specialists, including dermatologists and gynecologists, who could not identify a cause for her symptoms or offer an effective treatment. Gynecologists could identify no source on detailed gynecological and pelvic examination. Dermatologists could not find any skin-related problem; however, one doctor did prescribe the patient antifungal medication, which she stopped using after a few weeks due to no improvement in her symptoms. She had a poor quality of life due to the constant pain affecting her daily life activities. She spent most of her time inside the house and avoided social gatherings with the fear of having an episode of intense discomfort in public.

The reason for publishing this case is that no such case has been reported in the medical literature from Pakistan, which is a conservative Muslim country. There has been only one case report from a neighbouring country, India [8]. We must empathize with the patient, who endured immense pain and discomfort with her condition for 11 years, with countless visits to different specialists without any benefit due to the lack of understanding and knowledge of this disorder in the medical community. She also suffered from psychological trauma due to the shame and guilt involved with her symptoms that adversely affected her life. Finally, the response to dopaminergic drugs was dramatic and beneficial, allowing the patient to continue her daily life with ease.

The main aim of reporting this case is to create awareness among physicians and other members of the medical community about the value of a focused history and the need for an early neurological consult in such cases.

## Conclusions

This first documented case report of RGS from Pakistan, an LMIC country, provides unique perspectives about this disturbing and painful condition. It resulted in a poor quality of life and social isolation of this patient for 11 years due to lack of an accurate diagnosis. It can be effectively treated once a correct diagnosis has been established. This case reports also highlights the need to develop neurology in the LMIC, as many similar cases can remain undiagnosed for a long period due to the lack of expert neurological consultation. There is a need for further large-scale research on this condition and its possible variants. There is also a need to establish clear diagnostic criteria to make the diagnosis easy.
